# Coordinated Return-to-Work model reduces sickness absences after hip or knee arthroplasty: a registry-based study

**DOI:** 10.1007/s00402-026-06312-5

**Published:** 2026-04-17

**Authors:** Pauliina Kangas, Konsta Pamilo, Satu Soini, Maria Hirvonen, Visa Kervinen, Marja-Liisa Kinnunen

**Affiliations:** 1 Finnish Institute of Occupational Health, Helsinki, Finland; 2 Mehiläinen Hospital, Jyvaskyla, Finland; 3 Occupational Health Services, Terveystalo Ltd, Inari, Finland; 4 The Wellbeing Services County of Central Finland, Jyvaskyla, Finland; 5 School of Medicine, University of Eastern Finland, Kuopio, Finland

**Keywords:** Osteoarthritis, Occupational health services, Work ability

## Abstract

**Introduction:**

Working-age adults increasingly undergo total hip (THA) and knee (KA) arthroplasties. We evaluated how post-arthroplasty referral to occupational health services (OHS) using the Coordinated Return-to-Work (CRTW) model, affects return to work (RTW).

**Materials and methods:**

The CRTW model was evaluated through a benchmarking controlled trial. We used the electronic records of four hospitals in Finland to identify working-age THA and total/unicondylar KA patients before (control group, *N* = 668) and after (intervention group, *N* = 536) the CRTW model was implemented. We combined these data with sickness benefits registry data. The differences between the study groups’ RTW were analyzed using a Cox regression model, adjusting for age, sex, body mass index, number of special reimbursement entitlements for medicines, and earnings as covariates. Subgroup analyses included intervention participants whose sick leaves were prescribed by a surgeon according to the CRTW protocol.

**Results:**

After THA, the control group’s mean RTW-duration was 87.8 days, compared to 74.9 days for the intervention group, with the mean difference being 12.9 days (95% CI 5.7–20.2). The intervention group was associated with earlier RTW; HR 1.35 (95% CI 1.13–1.61). After KA, the control group’s mean RTW-duration was 107.8 days, while the intervention group’s was 93.4 days, with the mean difference being 14.4 days (95% CI 5.9–22.9), HR 1.29 (95% CI 1.09–1.54). In the subgroup analyses, the mean RTW-duration in the specifically targeted intervention groups was 60.2 days after THA and 84.7 days after KA. The mean differences compared with the control groups were 27.6 days (95% CI 19.1–36.1), HR 2.96 (95% CI 2.22–3.95) and 23.1 days (95% CI 10.9–35.3), HR 1.51 (95% CI 1.18–1.95), respectively.

**Conclusions:**

The CRTW model effectively shortens the RTW-duration after arthroplasty. Success depends on surgeons prescribing brief sick leaves and referring patients to OHS for work ability assessment.

## Introduction

The burden of osteoarthritis (OA) on healthcare resources is high and rising due to population growth, ageing and the obesity epidemic [1]. This has resulted in an increase in total hip and knee arthroplasties (THA and TKA) in the Western countries [2–6].

Successful postoperative recovery is crucial to reduce human affliction, save healthcare resources, and lessen the financial burdens on society. Fast-track protocols aim to enhance recovery [7–9], but recovery is mostly measured by organization-centred outcomes, such as length of stay in hospital (LOS) rather than by long-term recovery or work ability. In Finland, over 30% of TKA and THA are performed on people of working age, highlighting the importance of return to work (RTW) after surgery [10–16]. Studies indicate that 94% of hip and 87% of knee surgery patients in Finland RTW within a year. However, the RTW times vary in different countries, and work ability may be affected two years later [11–16]. Collaboration between surgical units and occupational health services (OHS) is essential to ensure optimal recovery and minimize unnecessary work absences. In the Netherlands, the Back At work After Surgery (BAAS) model was found to be more effective than usual care in improving RTW after KA [17]. In Finland, post-surgery consultations with OHS has previously remained rare although 90% of Finnish employees have access to statutory OHS [18]. Therefore, a Coordinated Return-to-Work (CRTW) model has been developed, which includes short hospital-prescribed sickness leave and systematic referral to OHS. By integrating fast-track protocols with occupational health strategies, the CRTW model supports holistic recovery and RTW, emphasizing not only medical recovery but also workplace adaptations and psychosocial support, which can reduce the time to RTW [19–20]. However, the previous Finnish registry study [20], may have underestimated the effects of the CRTW on RTW by evaluating the entire working-age population, rather than focusing only on patients eligible for OHS.

This registry study evaluated how effective the CRTW model is in terms of RTW in a population eligible for OHS using a benchmarking controlled trial [21].

## Methods

### Coordinated Return-to-Work (CRTW) model

**Fig. 1:**
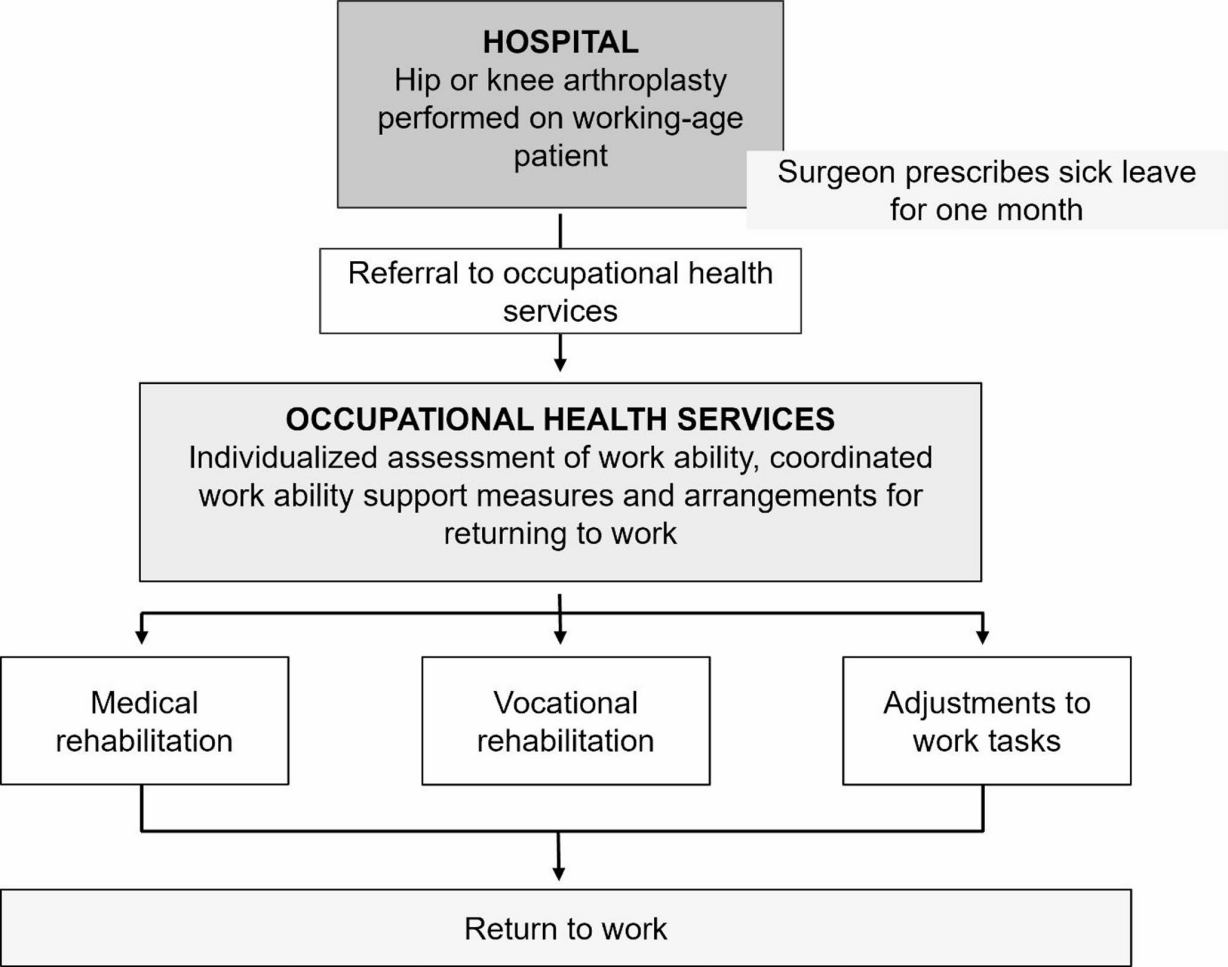
The principle of the CRTW-model in hip and knee arthroplasty patients. (Modified from previously published article [19])

The goal of the CRTW model is to individually assess work ability and effectively support RTW after hip or knee arthroplasty and it is aimed at patients who are eligible to use OHS. In the CRTW model, the surgeon prescribes the patient a one-month sick leave after the arthroplasty and refers them to OHS. The adequate total time to RTW is assessed in OHS, taking into account the demands of the work and whether the workload can be adjusted. RTW may be supported by guidance from an occupational physiotherapist, and an occupational health specialist can refer patients to various kinds of rehabilitation after arthroplasty. The occupational health negotiation is a commonly utilized tool, in which the OHS representatives, the employer and the employee participate. RTW is planned during the negotiation, and possible adjustments to work tasks are considered to facilitate successful RTW. Multifaceted arrangements made in OHS and in the workplace to support RTW after arthroplasty have been previously reported [19]. Figure 1 presents the principle of the CRTW.

### Study design

We used a benchmarking controlled trial [21] to assess the effectiveness of the CRTW model in terms of RTW after THA or knee (total or unicondylar) arthroplasty (KA).

### Setting and study population

We identified working-age patients (aged 25–62 years) who underwent THA or KA at four hospitals across three districts in Finland, using the data of electronic medical records (EMRs). The hospitals represented a range of settings, from a small local hospital to large university hospitals. Data were collected during two periods: November 2017 to October 2018 (pre-implementation) and in 2021 (post-implementation of the CRTW model). Because the CRTW model is designed only for patients covered by OHS, we identified eligible individuals by linking data from the Finnish Centre for Pensions’ Earnings Register with EMRs. This approach allowed us to determine which patients had pension-insured employment, indicating access to employer-provided OHS. Finnish legislation mandates employers to arrange OHS for all employees. However, unemployed working-age individuals and most entrepreneurs are not covered by these services, necessitating this additional step in identifying the target population.

**Fig. 2:**
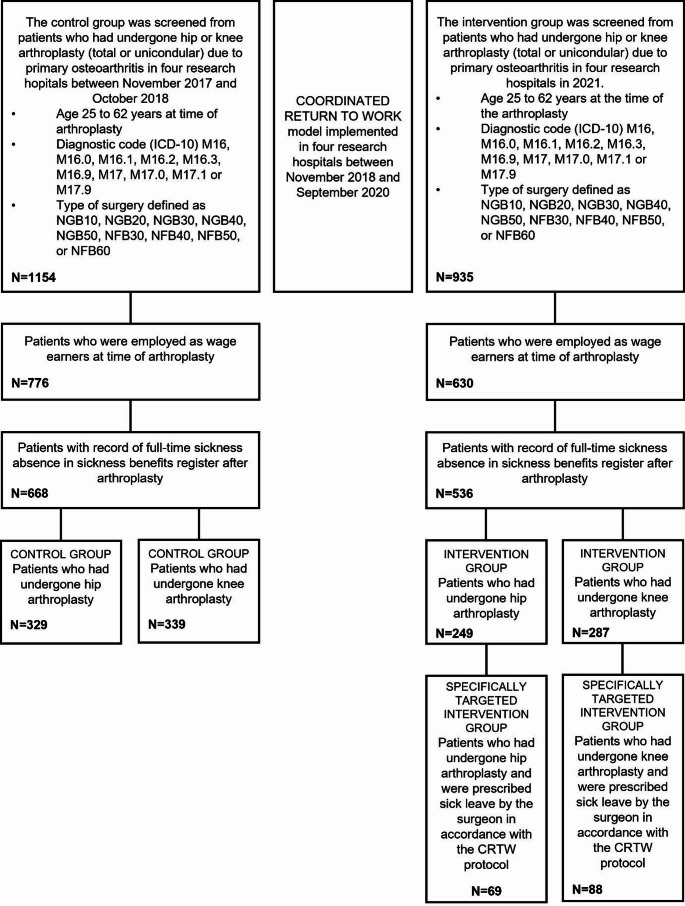
Flow chart of study participants

The intervention group consisted of patients who underwent THA or KA in 2021, while the control group included those who had surgery between November 2017 and October 2018. All patients underwent arthroplasty due to primary OA. Patients with diagnosed developmental dysplasia of the hip (DDH) were also included, due to variability in the coding of mild DDH and primary OA. Detailed information on diagnostic codes (ICD-10) and surgical procedure codes (NOMESCO) is provided in Fig. 2.

The whole study population was analyzed to answer the main study question, using an intention-to-treat (ITT) approach, which did not require precise information on whether the CRTW model was actually realized in all patient cases.

### Subgroup analysis

Clinical experience has shown that, despite the intended implementation of the CRTW model, human factors — such as lack of awareness of the protocol or oversight by surgical clinic staff — can result in the model not being applied consistently. To address this and strengthen the assessment of the CRTW model’s effectiveness on RTW timing, we performed subgroup analyses focusing on patients whose sick leave was prescribed by the surgeon in accordance with the CRTW protocol.

Data on the duration of surgeon-prescribed sick leave were available for 237 out of 536 patients, representing 44% of the intervention group. For 157 patients, sick leave was prescribed by the surgeon in accordance with the CRTW protocol. These patients formed a targeted intervention subgroup, which was compared to the same control group used in the main analysis.

### Variables

Time to RTW as a main outcome was defined by the date of the arthroplasty and the last day of sickness absence. We gathered information on sickness absences from the National Sickness Benefits Register maintained by the Social Insurance Institution of Finland. Because sickness benefits from the Social Insurance Institution of Finland are only granted after 10 days of sickness absence (the day when work disability is diagnosed plus 9 weekdays), we used the date of the arthroplasty as the first day of sickness absence. Sickness benefit periods that began within 15 days of discharge were included, and the diagnostic codes of the sickness absences had to correspond to the diagnosis for the surgery. For assessing sustained RTW, consecutive sickness absences with the same diagnostic code and a maximum of a 15-day gap between the periods were counted as the same sickness absence. Successful RTW meant returning to full-time or part-time work.

Demographic variables included age, sex, and body mass index (BMI) from the EMRs, annual earnings from Earnings Register of the Finnish Centre for Pensions, and special reimbursement entitlements for medicines from the register of the Social Insurance Institution of Finland, providing a proxy measure for chronic diseases [22].

### Statistics

The characteristics of the control group and the intervention group were compared using an independent samples t-test in cases of continuous variables, that is, age, BMI and annual earnings. The differences between the proportions of females/males and in the numbers of special reimbursement entitlements for medicines were compared using the Pearson’s chi-square test. In the table, the normally distributed variables are represented as means and standard deviations (SD), and the categorical variables as number of cases and percentages.

Time to RTW was reported as means and SDs, and in the figures as means and 95% confidence intervals (CI). The mean difference between the time to RTW of the study groups was also reported as CI.

The effect of the intervention group on time to RTW was examined using survival analysis. Kaplan–Meier survival curves were first generated to provide a visual comparison of the crude cumulative probability of RTW between the intervention and control groups. Cox proportional hazards regression analyses were conducted to test the statistical significance of earlier RTW probabilities in the intervention groups. The multiple Cox regression model included the group variable as well as demographic factors (age and sex) and baseline characteristics (BMI, annual earnings, and the number of special reimbursement entitlements for medicines) as covariates. This Cox model estimates the hazard ratio (HR), representing the relative probability of earlier RTW in the intervention group compared to the control group, while adjusting for confounding factors. Separate analyses were conducted for patients who underwent THA and KA.

The same analyses were also conducted for the subgroups. The subgroup analysis examined the differences between the control group and the specifically targeted intervention group, that is, the group that was prescribed sick leave by the surgeon in accordance with the CRTW protocol.

All testing was two-sided, and p-values of < 0.05 were considered statistically significant. We analysed some of the data using SPSS version 29.0 (IBM Corp, Armonk, NY). Kaplan–Meier curves were generated, and a Cox proportional hazards model was fitted using RStudio (Rstudio Team, 2024).

In a previous Finnish study on the CRTW model [20], an effect size of 0.435 was achieved with group sizes of 73 and 61, yielding an 80% power. Based on that power analysis, the group sizes in the current study are sufficient to ensure adequate statistical power.

## Results

### Study population

Altogether 2089 people met the inclusion criteria, of whom 1406 were employed as wage earners at the time of the arthroplasty, and 1204 (final study population) had a record of full-time sickness absence in the sickness benefits register after their arthroplasty. In the control group, 329 patients had undergone THA, and 339 patients had undergone KA (47 patients, 14% unicondylar arthroplasty (UKA)). In the intervention group, 249 patients had undergone THA, and 287 patients had undergone KA (31 patients, 11% UKA). Figure 2 shows the study population formation.

Table 1 presents the general characteristics of the study population. The control and the intervention groups did not differ in terms of age, sex, BMI or number of special reimbursement entitlements for medicines (*p* > 0.05 for all). Annual earnings were higher in the intervention group than in the control group among the KA patients (*p* = 0.01), while among the THA patients, the earnings did not differ between the intervention and the control groups (*p* = 0.56).

**Table 1:** Characteristics of study population

Characteristics	Hip arthroplasty			Knee arthroplasty		
	Control group, *N* = 329	Intervention group, *N* = 249	*P*-value	Control group, *N* = 339	Intervention group, *N* = 287	*P*-value
Age (years)	55 (5)	55 (6)	0.34	57 (4)	57 (4)	0.32
Sex			0.10			0.42
Female (N/%)	174 / 53%	149 / 60%		222 / 66%	179 / 62%	
Male (N/%)	155 / 47%	100 / 40%		117 / 35%	108 / 38%	
BMI (kg/m^2^)	28.4 (4.9)	28.8 (5.2)	0.35	31.4 (5.2)	31.1 (4.8)	0.49
Annual earnings about 2 years before arthroplasty (Euros)	46762 (27272)	45471 (25277)	0.56	36866 (18277)	40816 (20406)	*0.01*
Number of special reimbursement entitlements for medicines (N/%)			0.82			0.58
0	231 / 70%	178 / 72%		217 / 64%	175 / 61%	
1	65 / 20%	50 / 20%		84 / 25%	82 / 29%	
2 or more	33 / 10%	21 / 8%		38 / 11%	30 / 11%	

Values are means (standard deviations), except for the values for sex and the number of special reimbursement entitlements for medicines, which are shown as the number of cases and percentages. P-values denote differences between the control and intervention groups. BMI value was missing in 173 cases (14% of cases), and the information on annual earnings was missing in four cases (0.3% of cases)

*BMI* body mass index

### Time to return to work

**Fig. 3:**
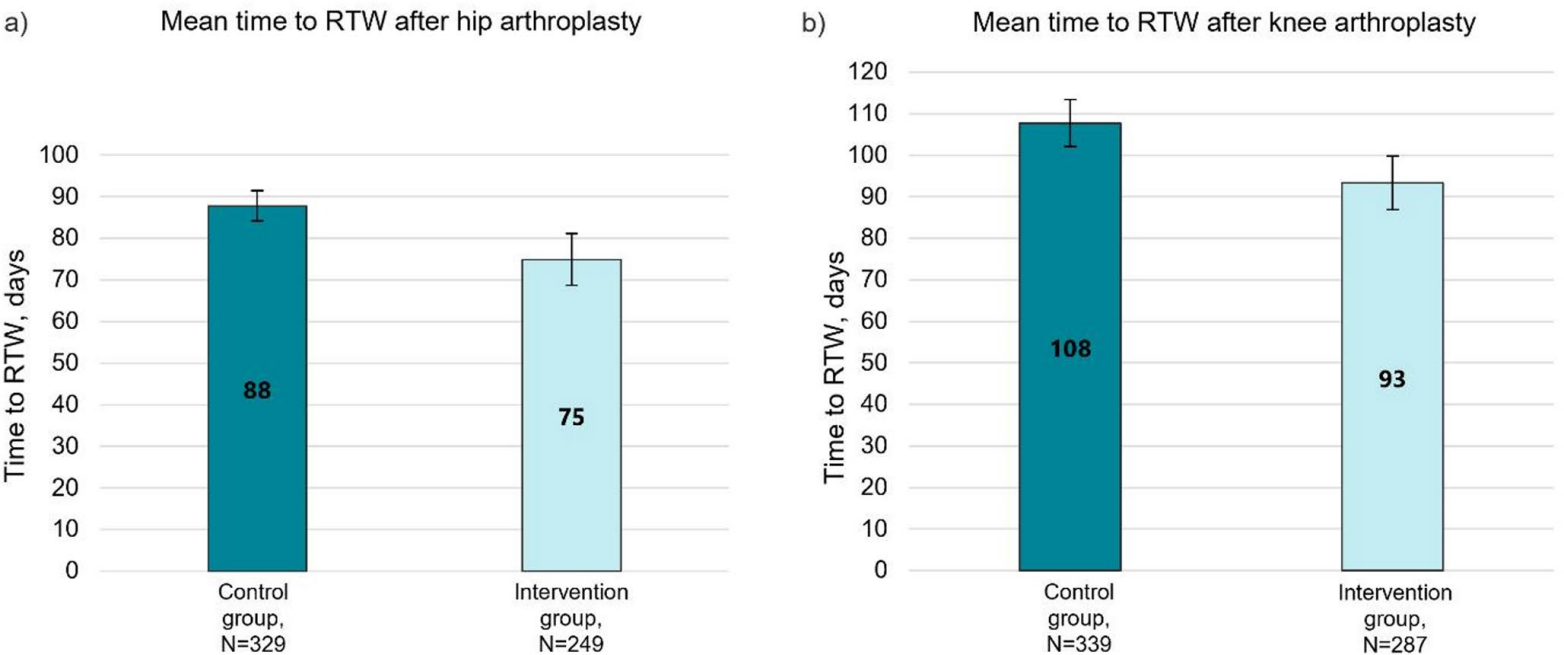
Time to return to work (RTW) after THA (**a**) and KA (**b**) in the study groups: control group and intervention group. Mean and 95% confidence interval

**Fig. 4:**
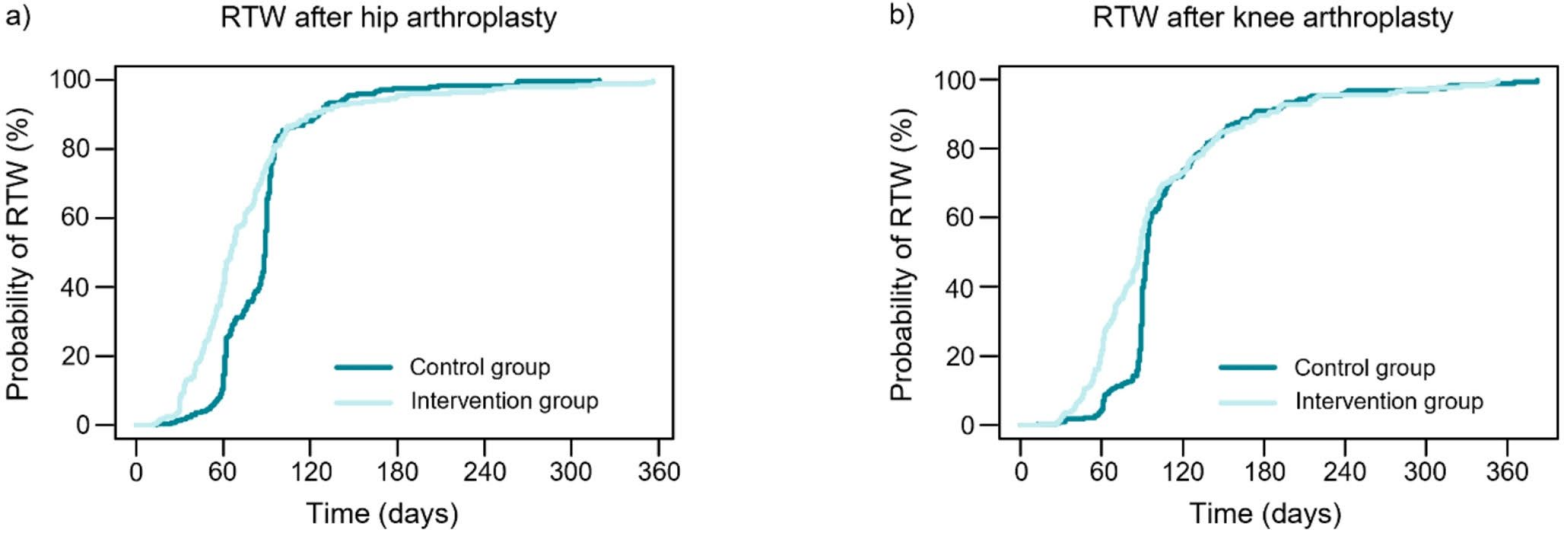
The Kaplan-Meier curves showing the probability of RTW over time for patients with THA (**a**) and KA (**b**) in the study groups: control group and intervention group

Among the THA patients, the mean time to RTW was 87.8 days (SD 34.1) in the control group, and 74.9 days (SD 50.0) in the intervention group (Fig. 3a). The mean difference between the time to RTW of the groups was 12.9 days (95% CI 5.7 to 20.2). The intervention group had a significantly higher hazard ratio for earlier RTW based on a Cox regression model adjusted for age, sex, BMI, number of special reimbursement entitlements for medicines, and annual earnings as covariates; HR = 1.35 (95% CI 1.13 to 1.61, *p* = 0.001). The Kaplan-Meier curve showing the probability of RTW over time is provided in Fig. 4a.

Among the KA patients, the mean time to RTW was 107.8 days (SD 52.7) in the control group, and 93.4 days (SD 55.7) in the intervention group (Fig. 3b). The mean difference between the time to RTW of the groups was 14.4 days (95% CI 5.9 to 22.9). The intervention group had a significantly higher adjusted hazard ratio for earlier RTW, with HR = 1.29 (95% CI 1.09 to 1.54, *p* = 0.003). Kaplan-Meier curve showing the probability of RTW over time is provided in Fig. 4b.

Among the UKA patients, the mean time to RTW was 87.6 days (SD 27.6) in the control group, and 94.3 days (SD 63.6) in the intervention group, with the number of patients being 47 and 31, respectively. Among the TKA patients, the mean time to RTW was 111.0 days (SD 55.0) in the control group, and 93.2 days (SD 54.8) in the intervention group, with the number of patients being 292 and 256, respectively.

### Subgroup analyses

**Fig. 5:**
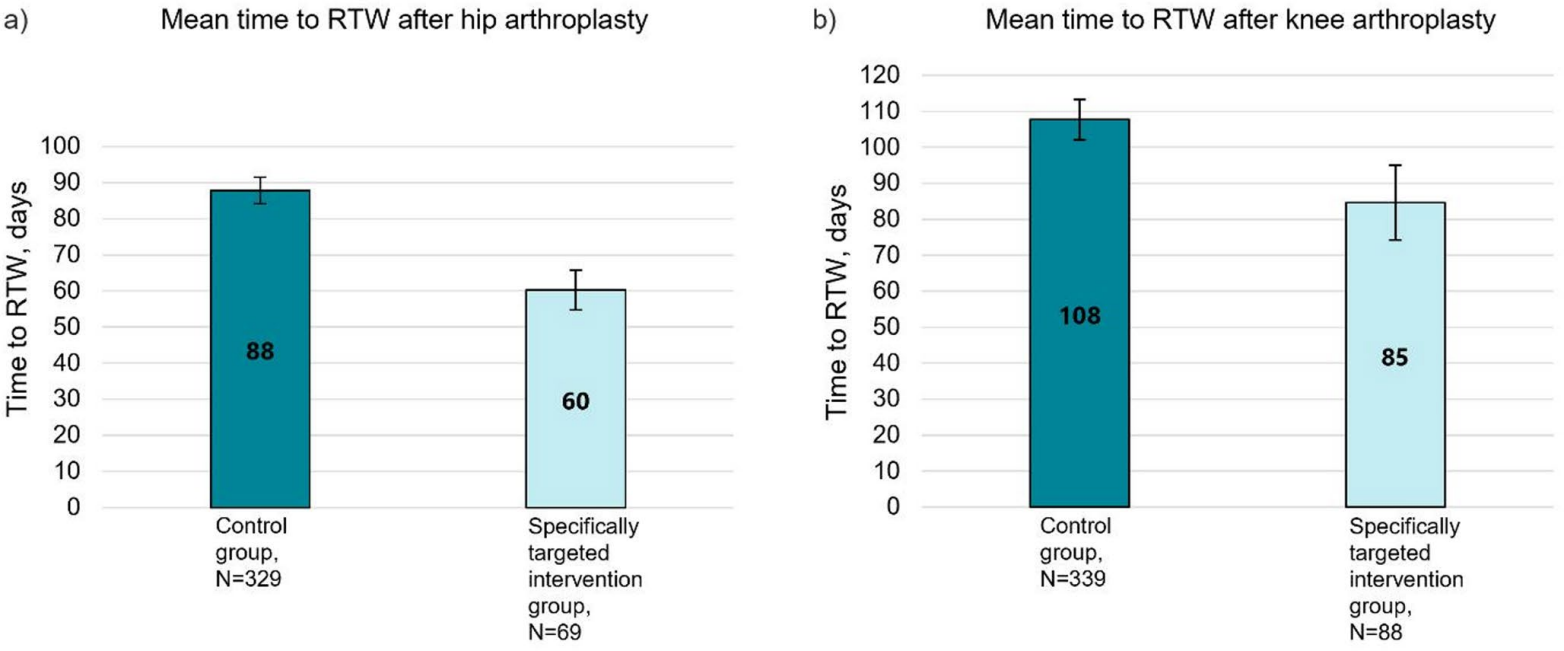
Time to return to work (RTW) after THA (**a**) and KA (**b**) in the study groups: control group, and specifically targeted intervention group (patients who were prescribed sick leave by the surgeon in accordance with the CRTW protocol). Mean and 95% confidence interval

**Fig. 6:**
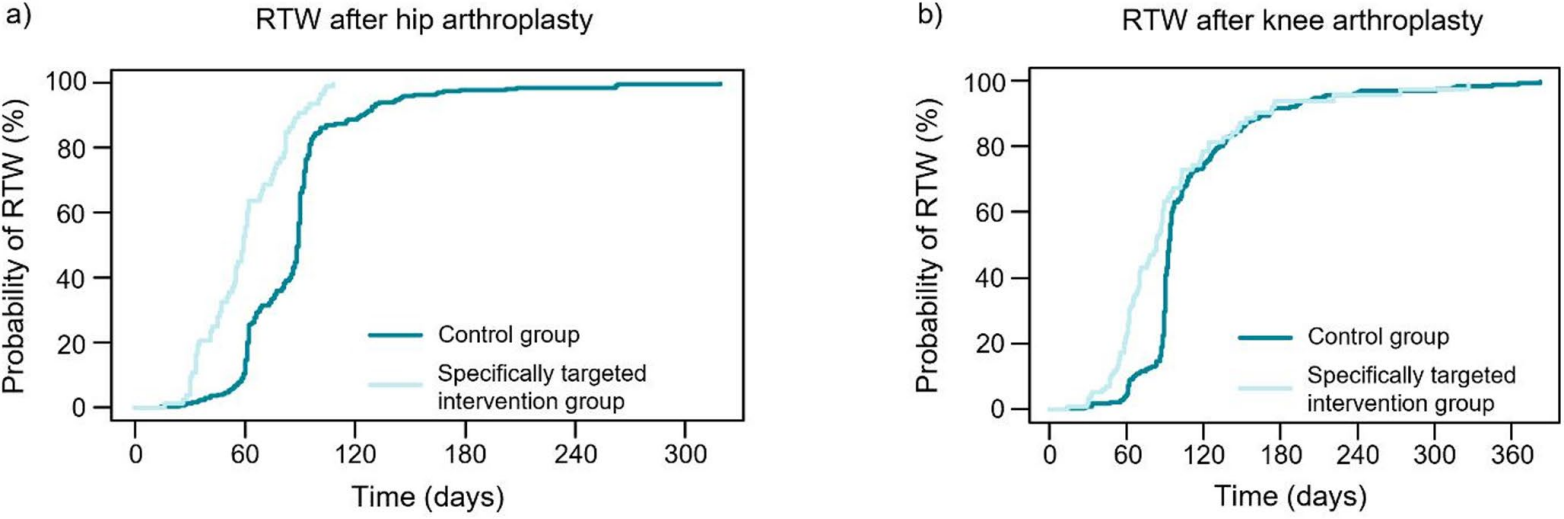
The Kaplan-Meier curves showing the probability of RTW over time for patients with THA (**a**) and KA (**b**) in the study groups: control group, and specifically targeted intervention group (patients who were prescribed sick leave by the surgeon in accordance with the CRTW protocol)

Among the THA patients with a surgeon-prescribed one-month sick leave in accordance with the CRTW protocol, the mean time to RTW was 60.2 days (SD 22.9) (Fig. 5a). The mean difference between the time to RTW of the control group and this specifically targeted intervention group was 27.6 days (95% CI 19.1 to 36.1). The specifically targeted intervention group had a significantly higher adjusted hazard ratio for earlier RTW, with HR = 2.96 (95% CI 2.22 to 3.95, *p* < 0.001). Kaplan-Meier curve showing the probability of RTW over time is provided in Fig. 6a.

Among the KA patients with surgeon-prescribed sick leave in accordance with the CRTW protocol, the mean time to RTW was 84.7 days (SD 49.3) (Fig. 5b). The mean difference between the time to RTW of the control group and this specifically targeted intervention group was 23.1 days (95% CI 10.9 to 35.3). The specifically targeted intervention group had a significantly higher adjusted hazard ratio for earlier RTW, with HR = 1.54 (95% CI 1.18 to 1.95, *p* = 0.001). The Kaplan-Meier curve showing the probability of RTW over time is provided in Fig. 6b.

## Discussion

### The main findings of the study

In this study, we evaluated the effectiveness of the CRTW model on the time to RTW after THA and KA, and showed that the model effectively shortens sickness absences after THA and KA. Subgroup analyses were performed on patients who were prescribed sick leave by the surgeon in accordance with the CRTW protocol, and these analyses showed that the effect of the CRTW model on RTW was even stronger.

The differences in results between the whole population and the subgroup suggest that the CRTW model is not systematically utilized within the intervention group of the entire population, and that the best effect of the CRTW model depends on whether the surgeon issues an initial one-month sick leave and then refers the patient to OHS. During this one-month period, the potential for RTW should be individually assessed by occupational health physician. The study results are in good concordance with the results of earlier register-based and survey studies [19–20], strengthening the evidence of this intervention. Even better outcomes can be anticipated as adherence to the model improves and its application becomes more consistent among all surgeons. Education and routines for monitoring activities in orthopedic units will facilitate the success of the CRTW model. It is important for each unit to have a designated person responsible for overseeing the CRTW model.

A previous study has shown that patients RTW earlier after UKA than after TKA [23]. In our study, the mean time to RTW was indeed shorter among UKA patients in the control group; however, in the intervention group, UKA patients did not RTW earlier than TKA patients. The number of patients who underwent UKA was relatively small, which did not allow for a more in-depth analysis of this phenomenon. However, the differences in recovery between UKA and TKA patients do not account for the main results of the study.

### The role of the CRTW model in shortening time to RTW

Referring patients to OHS after arthroplasty supports work ability more effectively. An individualized approach to work ability assessment is possible in OHS, where professionals can determine the appropriate time for RTW after arthroplasty taking into account not only the operation from which the patient is recovering, but also the demands of the work. OHS operate closely connected to workplaces, and to ease RTW, work tasks or work time can be modified in collaboration with the workplaces. The expertise of an occupational physiotherapist may be utilized, and a patient can also be referred to various kinds of rehabilitation through OHS to support their RTW. In the previous study, multiple RTW support measures were performed in OHS and in the workplaces after the patients had been referred to OHS. Occupational health negotiations were arranged in half of the patient cases following surgery. In about one out of five cases, the RTW process was facilitated by partial sickness allowance. The most commonly utilized workplace adjustments included allowing remote work, limiting work tasks, and increasing opportunities for breaks [19]. 

The roles of OHS and workplaces in RTW following arthroplasty have been evaluated in several studies. Employees have considered workplace support and job modifications important for easing RTW [24]. Additionally, the involvement of OHS professionals has been noted as a factor associated with a positive RTW experience [24]. Employers have also regarded OHS as an important partner in assisting with RTW after arthroplasty [25]. Collaboration between occupational physicians and orthopedic surgeons has been suggested to enhance RTW after KA [26]. The BAAS model, developed in the Netherlands, integrates medical and occupational care after KA, and has been shown to improve RTW [17].

### RTW as an important factor when evaluating the results of arthroplasty

In Finland, the mean economic loss of 1 sickness absence day varies in different sectors from 213 to 1058 euros [27]. According to the results of the subgroup analysis in our study, the number of sick leave days was reduced by an average of 28 days after THA and 23 days after KA, representing a cost effect ranging from thousands to tens of thousands of euros per patient. Therefore, the economic significance of the CRTW model is evident. Returning earlier to work is often also financially profitable for the patient, and preventing prolonged sickness absences may also improve long-term work ability and employment. The Dutch study has shown that the work-integrated care pathway, BAAS, also has a favorable economic impact [28].

Recovery from THA or KA is commonly assessed on the basis of organization-centred outcomes, such as LOS [9], or several functional performance and patient-reported outcome measures [29]. However, RTW is a significant outcome and has a considerable effect on patients’ social inclusion and the financial burden associated with osteoarthritis and arthroplasty. Consequently, assessments of recovery from arthroplasty among the working-age population should always include RTW.

### Strengths

The strength of this study is its relatively extensive data from multiple Finnish registers. The study population consisted of patients who underwent arthroplasty in different sizes of hospitals, and from a wide geographical area in Finland. In addition to the many hospitals that participated in this study, numerous OHS providers were involved in the CRTW model, and therefore the study provides comprehensive information on RTW after THA or KA. This study supports the previously observed effectiveness of the CRTW model. Another strength is its design, which enabled a study population of who were eligible to use OHS. The subgroup analyses further examined patients who had presumably undergone arthroplasty according to the CRTW model. The final factor that strengthens the reliability of the study results is that they were adjusted for age, sex, BMI, number of special reimbursement entitlements for medicines, and annual earnings.

### Limitations

In the adjusted analyses, information on BMI was missing for 14% of the study population. Moreover, preoperative sick leave, the physical demands of the job, or patient expectations regarding time to RTW [19] could not be taken into account due to limited information in the registry data. We were not completely certain about the patients who had undergone arthroplasty in accordance with the CRTW-model. The subgroup analyses gave us a strong indication of it, but the information on surgeon-prescribed sick leaves was available from only 2 out of 4 hospitals. Nevertheless, we observed that the CRTW model had a clear effect on RTW in the whole study population, even if the population included patients who had not undergone arthroplasty in accordance with the CRTW protocol.

## Conclusion

Collaboration between surgical clinics and OHS providers is an efficient tool for shortening the time to RTW after THA or KA. For a successful outcome, it is important that the surgeon prescribes only a short sick leave, and that the patient is referred to OHS for an individualized work ability assessment. The evidence of the effectiveness of the CRTW model promotes spreading the model widely into the field of orthopedic surgery.

## Data Availability

The research data were obtained from Findata, the Finnish Social and Health Data Permit Authority, with data permit THL/1415/14.02.00/2022. Findata was responsible for the pseudonymizing the data and ensuring anonymity in the final results. The datasets generated and analysed during the present study are not publicly available.
